# A Precise Prediction of the Chemical and Thermal Shrinkage during Curing of an Epoxy Resin

**DOI:** 10.3390/polym16172435

**Published:** 2024-08-28

**Authors:** Jesper K. Jørgensen, Vincent K. Maes, Lars P. Mikkelsen, Tom L. Andersen

**Affiliations:** 1 Department of Wind and Energy Systems, Technical University of Denmark, 4000 Roskilde, Denmark; 2 Bristol Composites Institute, University of Bristol, University Walk, Bristol BS8 1TR, UK; vincent.maes@bristol.ac.uk

**Keywords:** cure shrinkage, epoxy, finite element, UMAT, thermal expansion, volumetric shrinkage

## Abstract

A precise prediction of the cure-induced shrinkage of an epoxy resin is performed using a finite element simulation procedure for the material behaviour. A series of experiments investigating the cure shrinkage of the resin system has shown a variation in the measured cure-induced strains. The observed variation results from the thermal history during the pre-cure. A proposed complex thermal expansion model and a conventional chemical shrinkage model are utilised to predict the cure shrinkage observed with finite element simulations. The thermal expansion model is fitted to measured data and considers material effects such as the glass transition temperature and the evolution of the expansion with the degree of cure. The simulations accurately capture the exothermal heat release from the resin and the cure-induced strains across various temperature profiles. The simulations follow the experimentally observed behaviour. The simulation predictions achieve good accuracy with 2–6% discrepancy compared with the experimentally measured shrinkage over a wide range of cure profiles. Demonstrating that the proposed complex thermal expansion model affects the potential to minimise the shrinkage of the studied epoxy resin. A recommendation of material parameters necessary to accurately determine cure shrinkage is listed. These parameters are required to predict cure shrinkage, allow for possible minimisation, and optimise cure profiles for the investigated resin system. Furthermore, in a study where the resin movement is restrained and therefore able to build up residual stresses, these parameters can describe the cure contribution of the residual stresses in a component.

## 1. Introduction

Residual stresses in cured thermosets like epoxies, polyesters, and polyurethanes are inherent to the curing process. Residual stresses can lead to unwanted warpage, which can lead to issues during assembly, for instance in wind turbine blade the root sections [1]. Furthermore, crack growth, tunnelling cracks, and delamination are affected by residual stresses in composites [2]. In some scenarios, reduced mechanical performance is observed, i.e., the fatigue behaviour is reduced [3,4].

These cure-induced residual stresses can be directly coupled to the cure-induced strains from combined thermal and chemical shrinkage [5]. The chemical shrinkage is known to be related to the volumetric change the thermoset undergoes due to the polymerisation [6]. Bogetti and Gillespie [6] proposed a linear relationship between the degree of cure and the volumetric shrinkage. This was experimentally confirmed by Shah and Schubel [7] and Khoun et al. [8], using a rheometer to quantify the shrinkage. Later, a non-linear model was proposed [9] allowing for a more complex shrinkage behaviour of thermosets. This non-linear shrinkage was later observed by measuring the volumetric shrinkage with a gravimetric/dilatometric setup by Li et al. [10]. Recently, experimental observations showed that the volumetric shrinkage determined using density measurements could be used to estimate the chemical shrinkage within reasonable accuracy, applying a linear fit [5]. Even though there has been a lot of research in this field, it remains unclear which approach yields the most accurate relationship between the degree of cure and the volumetric shrinkage.

Several phenomenological models exist to quantify the cure development, or the degree of cure as it is often referred to [11,12]. Some of these models capture only the kinetics and reaction patterns of the thermoset mixture [11], while others include the diffusion-controlled behaviour stemming from the influence of the glass transition temperature on the reaction rate [12]. The most well-established model for the evolution of the glass transition temperature in relationship with the degree of cure was coined by Dibenedetto [13]. As this transition occurs, thermosets are known to suffer significant changes in properties and behaviour [14], influencing the cure shrinkage.

Thermal shrinkage has also been a topic of substantial research over the years, with a common method for measuring thermal expansion being Thermal Mechanical Analysis (TMA) [8]. Previous studies have used this method to investigate glassy polymers, including both thermosetting and thermoplastic systems [8,15,16]. One study of an epoxy system using TMA [8] proposed a model for the non-linearity of thermal expansion approaching and crossing glass transition temperature for cured samples. The same study demonstrated that the thermal expansion is a function of the degree of cure above the glass transition temperature. Studies have shown that thermal expansion could also be measured with Dynamic Mechanical Analysis [15] and that glassy polymers can depend on heating and cooling [15,16]. Korolev et al. [16] showed that the difference in thermal expansion coefficient between heating and cooling could lead to a variation in the strain observed, thus demonstrating that thermal expansion in thermosets is complex.

This current work studies the cure shrinkage of a neat (i.e., without fibres) epoxy resin type typically used in wind turbine blade manufacturing. A cure kinetic and glass transition temperature model are used based on parameters found for a specific resin system investigated by Jørgensen et al. [17]. These models are used in experimental trials where the resin is free to contract and expand, with no external loads applied. The experimental method applied was proposed by Mikkelsen et al. [5]. This method uses fibre optic sensors with Fibre Bragg Gratings (FBG) similar to that used in other studies [3,4,5,18,19,20]. A conventional model describing the evolution of the chemical shrinkage with the degree of cure is investigated in terms of how it reflects the chemical shrinkage observed in experiments. In addition, a novel complex thermal expansion model is proposed to relate thermal effects to the measured cure shrinkage. This material behaviour is implemented into a simulation framework that considers the chemical and thermal shrinkage as the governing constituents used by Jørgensen and Mikkelsen [21]. In the end, a procedure will be delivered to accurately predict the shrinkage of thermosetting epoxy resins and allow for realistic minimisation in reducing residual stresses.

## 2. General Theory

This section describes the equations and models applied for both experimental and numerical aspects of this study.

### 2.1. Cure Kinetic Model

A cure kinetic model [12] accounting for the interaction between the glass transition temperature, $T_{g}$, and the evolution of the degree of cure, *X*, is used to account for the reduced molecular mobility effect by diffusion control into the cure predictions, resulting in a model defined as:

(1) $$\begin{array}{c} \frac{dX}{dt}=\frac{K\left(T\right)X^{m}{\left(1-X\right)}^{n}}{1+\exp\left[C(X-X_{c}\left(T\right))\right]},\\ K\left(T\right)=A\exp\left(-\frac{e_{a}}{RT}\right),\\ X_{c}\left(T\right)=X_{cT}T+X_{c0}. \end{array}$$

The model incorporates the Arrhenius reaction equation, K(T), which consists of the preexponential factor, *A*, the activation energy, $e_{a}$, and the universal gas constant, *R*, and is commonly used to model the cure behaviour in epoxy resin systems. Furthermore, *n* and *m* are power law coefficients and in the denominator, the model is described by an exponential function which captures the reduction in rate of cure caused by the reduced molecular mobility at higher decrees of cure. This behaviour is governed by the diffusion constant, *C*, which captures how abruptly the cure reaction slows down and the critical degree of cure, $X_{c}$, which captures the degree of cure at which the polymer chains length and cross-links begin to prevent remaining reaction sites from meeting. This value depends on temperature through the glass transition temperature, $T_{g}$, as temperatures above $T_{g}$ result in higher molecular mobility, which facilitates the meeting of reaction sites, and therefore, delays the drop in reaction rate. The critical degree of cure is computed using the baseline critical degree of cure at a temperature of zero, $X_{c0}$, and the increase in the critical degree of cure per degree increase in temperature, $X_{cT}$. The degree of cure *X* is calculated through numerical integration (2) in time as:

(2) $$X=\int_{0}^{t}\frac{dX}{dt}dt.$$

The integrated *X* values, thus, depend on temperature, *T*, and time, *t*, relating the cure kinetic model to the specific curing profile applied to the studied epoxy resin.

### 2.2. Glass Transition Temperature

The DiBenedetto relation [13] in (3) relates *X* to the midpoint value of the $T_{g}$ range. The relation involves the final $T_{g}$ for a state of complete cure, $T_{g\infty }$, the initial $T_{g}$ for a state of zero cure, $T_{g0}$, and a fitting parameter, ξ, and represents the ratio of the segmental mobility of the fully cured polymer to that of the initial monomers under the assumption of constant lattice energies [22] in the form:

(3) $$T_{g}\left(X\right)=T_{g0}+\frac{\xi X(T_{g\infty }-T_{g0})}{1-(1-\xi )X}.$$

### 2.3. Cure-Dependent Load-Transferring Volumetric Shrinkage

In the experimental setup, shrinkage can only be measured if there is load transfer between the resin and the fibre optic sensor. The ability to carry a load is also needed for residual stresses to develop. Thus, the volumetric shrinkage of the resin in the liquid phase is ignored. The shrinkage model (4) only considers the load-transferring volumetric shrinkage, $V_{sh}$. To this end, the degree of cure at which the resin begins to transfer the load is denoted by $X_{\sigma }$, which is close to but not the same as the degree of cure at gelation, $X_{gel}$. The magnitude of $X_{\sigma }$ is found experimentally based on a strain tolerance of |ε|>0.005% from the optical fibre with FBG [5] and using the cure kinetic model with the thermal history measured using the thermocouple. This shrinkage model assumes that the shrinkage from the load transfer point until the end of the cure, $X_{end}$, results in the load-transferring volumetric shrinkage, $V_{sh}^{end}$. The shrinkage model is dependent on *X* through a second-order term Johnston [9] following the conditions:

(4) Vsh=0,Xσ≤X<XσVshendX−XσXend−Xσ2,Xσ≤X<XendVshend,Xσ≤X≥Xend.

The parameters used in the shrinkage model are determined in Section 6.

### 2.4. Complex Thermal Expansion

**Figure 1:**
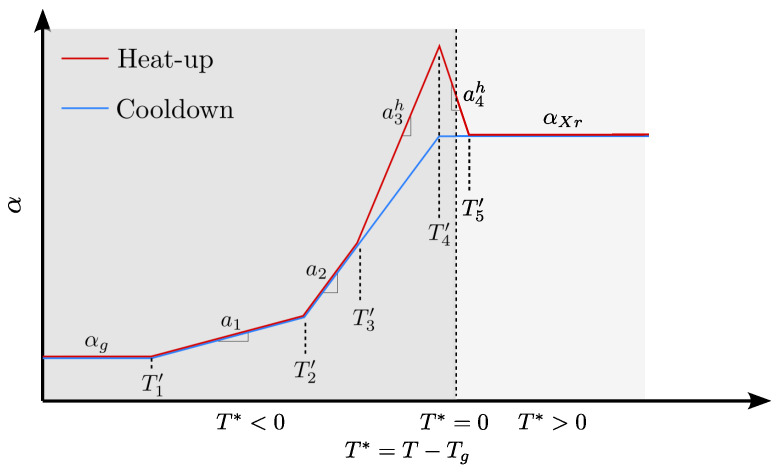
The thermal expansion model involving the transition for the difference between the instantaneous cure temperature *T* and the glass transition temperature, $T_{g}$.

The thermal expansion model extends an earlier model [8] by segmenting the thermal expansion development into several transitional stages. This modification was motivated by the observed behaviour, which was judged to be best captured using a piece-wise linear curve. The expanded model also captures heat-up- and cooldown-dependent behaviour [15] not previously present. The structure of the thermal expansion transition is shown in Figure 1.

The expanded model divides the thermal expansion into a thermal expansion for heat-up, $\alpha_{resin}^{h}$, and one for the cooldown, $\alpha_{resin}^{c}$. The expansion is related to the parameter $T^{*}$, which is the difference between the instantaneous temperature, *T*, and $T_{g}$. Before the load-transfer point, i.e., in the liquid phase, the thermal expansion of the resin is ignored (5) as it would not contribute to the cure-induced strain, and hence:

(5) $$\begin{array}{c} \alpha_{resin}^{h}=\alpha_{resin}^{c}=0,\;X<X_{\sigma }. \end{array}$$

Figure 1 shows the behaviour below the glass transition $T^{*}<0$ and after the glass transition temperature is passed $T^{*}>0$. Far from the glass transition temperature, below $T_{1}^{\prime }$, the thermal expansion is constant, but above $T_{1}^{\prime }$ and through $T_{2}^{\prime }$, $T_{3}^{\prime }$, and $T_{4}^{\prime }$, the thermal expansion increases, still in the glassy regime as $T^{*}<0$. Below $T_{3}^{\prime }$, the heat-up and cooldown increase in thermal expansion is described by $a_{1}$ and $a_{2}$. Above $T_{3}^{\prime }$, it becomes important to distinguish between heat-up and cooldown. The increase in thermal expansion during heat-up follows $a_{3}^{h}$. This is followed by a decrease in thermal expansion $a_{4}^{h}$ across the glass transition temperature from $T_{4}^{\prime }$ to $T_{5}^{\prime }$. During heat-up, the thermal expansion is constant relative to $T_{g}$ above $T_{5}^{\prime }$, and similarly, during cooldown from $T_{4}^{\prime }$. In both cases, the thermal expansion depends on the degree of cure when the glass transition is exceeded [8] and denoted $\alpha_{Xr}$. The relationship with the degree of cure follows a parabolic development. The equations describing the thermal expansion development during heat-up are as follows:

(6) αresinh=αg,T1′≤T*<T1′a1(T*−T1′)+αg,T1′≤T*<T2′a2(T*−T2′)+a1(T2′−T1′)+αg,T2′≤T*<T3′a3h(T*−T3′)+a2(T3′−T2′)+a1(T2′−T1′)+αg,T3′≤T*<T4′a4h(T*−T4′)+a3h(T4′−T3′)+a2(T3′−T2′)+a1(T2′−T1′)+αg,T4′≤T*<T5′αXr,T4′≤T*≥T5′.

Similarly to the set of equations describing the heat-up (6), a set of equation prevail for the cooldown (7):

(7) αresinc=αg,T1′≤T*<T1′a1(T*−T1′)+αg,T1′≤T*<T2′a2(T*−T2′)+a1(T2′−T1′)+αg,T2′≤T*<T4′αXr,T4′≤T*≥T4′.

The parabolic equation describes the cure-dependent thermal expansion $\alpha_{Xr}$ in (8) as:

(8) $$\alpha_{Xr}=a_{X2}X^{2}+a_{X1}X+a_{X0}.$$

The parameters describing the increase in the cure-dependent thermal expansion are $a_{X2}$, $a_{X1}$ and $a_{X0}$. The thermal expansion parameters necessary in the proposed model (5)–(8) are fitted in Section 6.

## 3. Modelling Constituents

The following are the necessary constituents considered in the modelling used in this study.

### 3.1. Thermal Behaviour

The thermal behaviour applied (9) follows the energy balance equation [1]:

(9) $$\Delta U=c_{p}\Delta T-H_{T}\frac{dX}{dt}\Delta t.$$

The incremental energy balance is described as ΔU for every time increment. The exothermal behaviour of the epoxy resin during curing is considered by including the total enthalpy of the reaction, $H_{T}$, multiplied by the cure rate and size of the time step. In addition, the resin density is as follows:

(10) $$\rho_{resin}=\rho_{resin}^{init}X+\left(X_{end}-X\right)\rho_{resin}^{end},$$

which is modelled using the rule of mixture between resin density in uncured state, $\rho_{resin}^{init}$, and cured state, $\rho_{resin}^{end}$, which were experimentally measured, see Section 6.

### 3.2. Mechanical Constituents

The constituents used for the mechanical behaviour are based on the constituents included in multiple studies [6,21]. The total, linear, cure-induced strain is taken as:

(11) $$\Delta \varepsilon^{tot}=\Delta \varepsilon^{ch}+\Delta \varepsilon^{th}.$$

The incremental thermal strain, $\Delta \varepsilon_{th}$, develops according to:

(12) $$\Delta \varepsilon^{th}=\alpha_{resin}\Delta T,$$

and the load-transferring incremental linear chemical strain, $\Delta \varepsilon_{ch}$, in the model is defined as the incremental isotropic change in the specific volumetric shrinkage following [6] with:

(13) $$\Delta \varepsilon^{ch}=\sqrt[{3}]{1+\Delta V_{sh}}-1.$$

Finally, the incremental volumetric shrinkage, $\Delta V_{sh}$, is defined by the volume change of a cubic element normalised by its original volume and is thus unitless.

The development of the cure-dependent load transferring shrinkage over time can be related to the incremental chemical strain in (13) by deriving (4) to incremental form by differentiation for *X* and *t* as in (14). Giving the volumetric shrinkage in the incremental form for the modelling perspective:

(14) $$\Delta V_{sh}=\frac{dV_{sh}}{dX}\Delta X\;;\;\;\Delta X=\frac{dX}{dt}\Delta t.$$

## 4. Experimental Method

### 4.1. Material System

In the present study, an industrially available thermoset epoxy resin is investigated. The resin is a conventional diglycidyl ether of bisphenol-A (DGEBA). The hardener is a modified cyclo-aliphatic- and aliphatic-amine. A mixing ratio by weight used is base: hardener; 100:31, following supplier guidance.

### 4.2. Reaction Mechanics

The parameters for the cure kinetics model (1) are based on the work performed in a previous study [17]. The study included the fitting and analysis of DSC data from this specific resin system. It finalised a set of cure parameters given in Table 1 together with the total enthalpy of the reaction given later in Table 6. The parameters will predict the degree of cure from (1) and the midpoint value of the glass transition temperature from (3).

### 4.3. Experimental Setup

**Figure 2:**
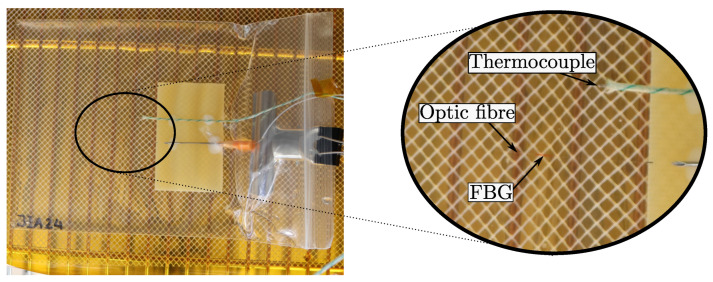
The experimental setup consists of a thin polymer bag. The specimen size is approx 150 × 150 mm and has an average thickness of 4 mm. A fibre optic sensor with an FBG and thermocouple is placed near the middle of the thickness.

The experimental setup applied in this study is equivalent to that used in a previous study [5] and similar to others [3,4,18,19,20]. The resin is in a stress-free state because there are no outer loads or constraining elements, allowing the resin to contract and expand and, therefore, to be considered unconstrained [5]. The setup shown in Figure 2 consists of a thin polymer bag, an optic fibre with Fibre Bragg Gratings (FBG), placed with a thermocouple inside the bag. The thermocouple monitors the temperature response during curing, and the FBG monitors the strain. The error of measurement from the optic sensor in a setup similar to this was discussed in the appendix of an earlier study [5]. Furthermore, the tail length of the optical sensor, from the FBG to the end of the sensor, is important for the accuracy of optical sensors [19]. It was found that the possible error from shear lag on the strain measured using this setup is negligible. As the tail length, $l_{f}$ of the optical sensor was well above $420r_{f}$, where $r_{f}$ is the optic fibre radius. This ratio was reported to give high sensibility even with low resin stiffness [19]. The resin is mixed, degassed, and injected into the polymer bag. Possible air entrapments during infusion are then removed from the bag. The dimensions of the neat resin after infusion are 150 × 150 mm and a thickness of 4 mm. The optic sensor and thermocouple are placed near the middle of the thickness.

## 5. Numerical Implementation

The numerical implementation was performed in the commercial finite element software Abaqus^®^2023. The applied material behaviour lies outside the boundaries of the built-in behaviour of the software. Therefore, the implementation used a user-defined material description through a FORTRAN programming-based subroutine offered by Abaqus^®^. This section gives a brief overview of the subroutines used and which parts of the models they were used to implement. A more detailed description and the actual subroutine can be obtained on request to the authors.

### 5.1. User-Defined Material Heat Transfer—UMATHT

The first part of the user-subroutine is the UMATHT, which handles the resin heat transfer and updates any changes in thermal properties. It is in this subroutine that the cure development is implemented. The main equations are the energy balance Equation (9), the degree of cure (1), the glass transition temperature (3), and the change in density (10).

### 5.2. User-Defined Expansion—UEXPAN

Coupled with the UMATHT, the subroutine UEXPAN is passed the necessary state variables from UMATHT to determine the thermal expansion (12) with framework from Figure 1 and the volumetric shrinkage (4) resulting in chemical strains (13). This results in the strain governed by (11).

## 6. Experimental Results

### 6.1. Cure Experiments

The cure profiles investigated with the setup explained in Section 4.3 are presented in Table 2. These profiles have been chosen to investigate the effects of different pre-cure temperatures and the effects of the length of the pre-cure on the resulting shrinkage. The notation of the naming follows that of a previous study [5]. The number refers to the cure temperature and the brackets [] denote the part of the cure profile considered the pre-cure, e.g., in $\left[40_{L}\right]80_{L}$, the [40] refers to 40 °C as the pre-cure isothermal temperature and 80 refers to an 80 °C isothermal post-cure. Additionally, the ${\left(\right)}_{L}$ stands for a long cure time of 8 h or more, the ${\left(\right)}_{M}$ stands for medium-length cure time, which is more than 2 h and less than 8 h, and ${\left(\right)}_{S}$ stands for a short cure time of 2 h or less.

**Figure 3:**
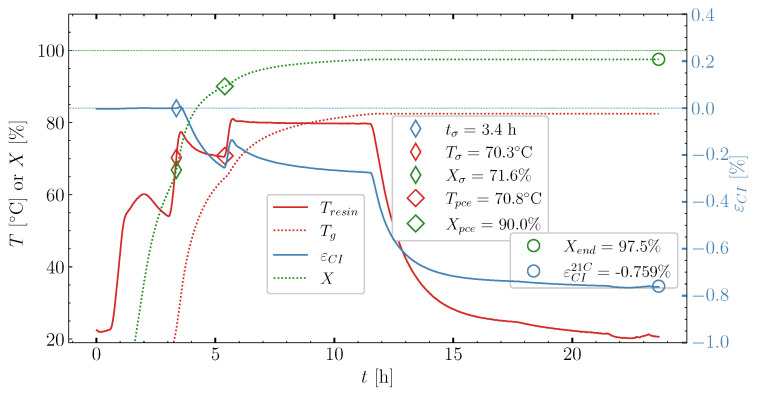
Cure experiment $\left[50_{S}70_{S}\right]80_{M}$—strain measured with the optic FBG and temperature developing in the oven $T_{oven}$, the temperature recorded inside the resin $T_{resin}$ by the thermocouple, as well as the degree of cure *X* and $T_{g}$ predicted based on the recorded resin temperature. The blue dotted lines represent zero strain. the green dotted line represents a level of 100% cure.

Figure 3 shows case $\left[50_{S}70_{S}\right]80_{M}$ and the resulting temperature and cure-induced strain monitored over the duration of the cure profile. The degree of cure and, subsequently, the glass transition temperature are predicted based on the models in (1) and (3), respectively. A strain tolerance [5] determines the load transfer point. Based on the load transfer point, the time, the degree of cure, and the temperature at which load transfer occurs are found. The index ${\left(\right)}_{\sigma }$ denotes the values at the load transfer point and in the plot it is denoted by narrow diamond-shaped points on the curves. Past the load transfer point, the points denoting the end of pre-cure ${\left(\right)}_{pce}$, shown by wide diamond points, are plotted on the temperature and the degree of cure curves. The value of the degree of cure at the pre-cure end, $X_{pce}$, will be used to evaluate the effect of pre-cure length on the measured cure-induced strain. When the resin has cooled to room temperature $T_{room}$ = 21 °C at the very end of the cure, the final cure-induced strain $\varepsilon_{CI}^{21^{\circ }C}$ is found together with the final degree of cure. At this instance, the cure-induced strain is $\varepsilon_{CI}^{21^{\circ }C}$= −0.759% and the corresponding degree of cure $X_{end}$ = 97.5%. The main results from the cases studied are compiled into Table 2 next to the cure profile parameters. The whole data figure set, like for the case illustrated in Figure 3, is available for download [23].

**Figure 4:**
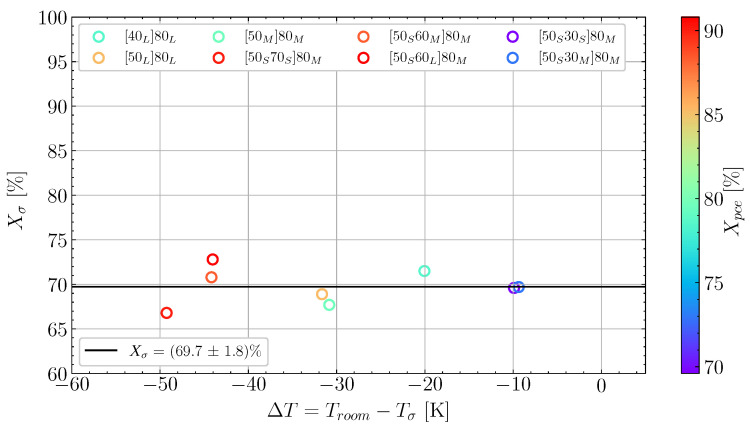
The degree of cure at the load transfer point, $X_{\sigma }$ recorded for the experiments in Table 2. As a function of the difference in temperature between the load transfer point and room temperature, ΔT. Colour-mapped according to the degree of cure at the end of pre-cure.

Based on data analysis of all the cure experiments listed in Table 2, Figure 4 shows $X_{\sigma }$ as a function of the temperature difference, ΔT, which is calculated as the difference between the temperature at the load transfer point, $T_{\sigma }$, and room temperature, $T_{room}$. Each case has a value of $X_{pce}$, which is colour-mapped across the investigated cases. This way, the plot demonstrates if the temperature and the length of pre-cure influence the degree of cure at the load transfer point. In the case of Figure 4, there is no obvious trend between ΔT and $X_{\sigma }$ or $X_{pce}$ and $X_{\sigma }$. Confirming that the parameter $X_{\sigma }$ should be independent of the cure temperature and the length of the pre-cure. Thus, the overall behaviour agrees with a previous study [5]. The average degree of cure at load transfer was $X_{\sigma }=69.7\%$, with a reasonably low variation.

**Figure 5:**
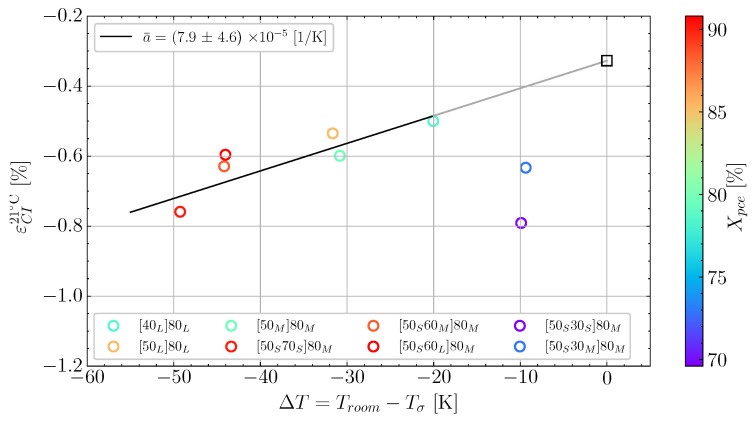
The final cure-induced strains measured at $T_{room}$ as function of ΔT and colour-mapped according to $X_{pce}$.

The cure-induced strain has been plotted as a function of the temperature difference ΔT in Figure 5 for the cases. It is observed that there is a substantial scatter in the measurements. However, by using a linear fit, a trend between the temperature difference and the strain can be observed. The two measurements at ΔT=−10 K have been excluded from the linear fit indicated by the grey line. This is due to these measurements seeming to be governed by other mechanisms. Therefore, the region from ΔT −20 K to 0 K is associated with some uncertainty. Hence, the grey-coloured trendline is used to demonstrate the region of uncertainty. The slope of $7.9\;\times \;10^{-5}$ K^−1^ is similar to the slope found by a previous study for a similar unconstrained resin [5]. As there is a larger scatter around the linear fit than in the previous study, it is relevant to study the effect of $X_{pce}$ for the different cases in Figure 5. The degree of cure at the pre-cure end, $X_{pce}$, seems to influence the cure-induced strain. If one observes the colour bar and the two measurements with ΔT≈−10 °C, there is a significant difference in $X_{pce}$ of around 5% reflected by the difference in colour. Similarly, for the two cases at ΔT≈−30 °C, the difference in colour on the measurements lead to 4% difference in $X_{pce}$.

**Figure 6:**
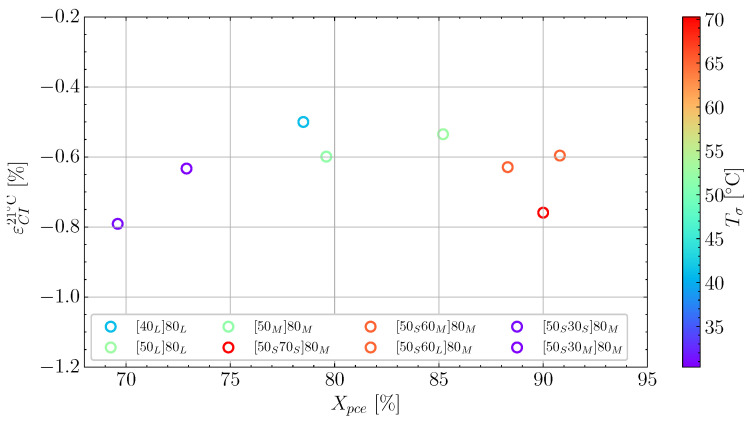
The relationship between $\varepsilon_{CI}^{21^{\circ }C}$ and $X_{pce}$, demonstrating the effect of pre-cure length on the measured strain.

To better clarify the influence of $X_{pce}$ on $\varepsilon_{CI}^{21^{\circ }C}$, Figure 6, shows the cure-induced strain $\varepsilon_{CI}^{21^{\circ }C}$ as a function of $X_{pce}$ with a colour-map represented by the load transfer temperature $T_{\sigma }$. The figure shows that the strain observed differs even with the same $T_{\sigma }$, i.e., points with the same colour. However, the $X_{pce}$ values on the horizontal axis differ for the same $T_{\sigma }$. Therefore, Figure 6 demonstrates a clear effect on the observed cure-induced strain with the evolution of cure past the load transfer point during pre-cure.

**Figure 7:**
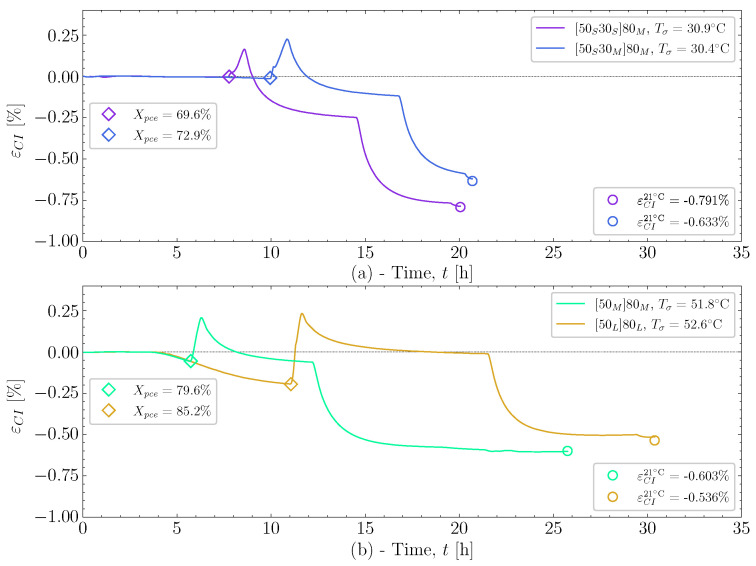
(**a**) The strain measured for case $\left[50_{S}30_{S}\right]80_{M}$ and $\left[50_{S}30_{M}\right]80_{M}$, demonstrating the effect of the length of pre-cure on these similar cases. (**b**) Strain measured for the cases $\left[50_{L}\right]80_{L}$ and $\left[50_{M}\right]80_{M}$ to show the effect pre-cure length.

This pre-cure effect is investigated in more detail with Figure 7 for cases that lead to differences in cure-induced strain. Four cases have been selected: Figure 7a show the two cases $\left[50_{S}30_{S}\right]80_{M}$ and $\left[50_{S}30_{M}\right]80_{M}$ with $T_{\sigma }\approx 30$ °C, and Figure 7b show two cases, $\left[50_{L}\right]80_{L}$ and $\left[50_{M}\right]80_{M}$ with $T_{\sigma }\approx 50$ °C. The colours applied for each case follow that of the colour-mapping for $X_{pce}$ applied in both Figure 4 and Figure 5.

In Figure 7a, it can be seen that the added time in $\left[50_{S}30_{M}\right]80_{M}$, and thereby higher $X_{pce}$, allows the resin to expand a little more than $\left[50_{S}30_{S}\right]80_{M}$ in the heat-up followed by the pre-cure. This is reflected in the observed final cure-induced strain, $\varepsilon_{CI}^{21^{\circ }C}$. The difference in expansion towards the post-cure between the two cases covers, for the most part, the difference in the cure-induced strain. Similarly, in Figure 7b, the longer pre-cure of the $\left[50_{L}\right]80_{L}$ case allows the resin to cure substantially more, resulting in a higher expansion relative to $\left[50_{M}\right]80_{M}$. Again, this results in a difference in the final cure-induced strain observed. Figure 7b also shows that the difference in pre-cure length affects the chemical strain, dominating the pre-cure and post-cure isothermals. For case $\left[50_{L}\right]80_{L}$, which has a substantially higher value of $X_{pce}$, the shrinkage at the end of the post-cure more or less has cancelled out, unlike $\left[50_{M}\right]80_{M}$, which is already in the negative strain regime at the end of post-cure. It is seen that the shrinkage during the pre-cure influences the total cure-induced strain by the end. This shrinkage is mostly chemical strain as the temperature changes are relatively small. The thermal expansion and the chemical shrinkage seem to relate to the magnitude of $X_{pce}$ when the resin is heated up for post-curing. A similar effect, as demonstrated in Figure 7, has been observed previously [5]. However, the resin system studied then seemed to be less susceptible to the effect of pre-cure length.

### 6.2. Determining Volumetric Shrinkage

**Figure 8:**
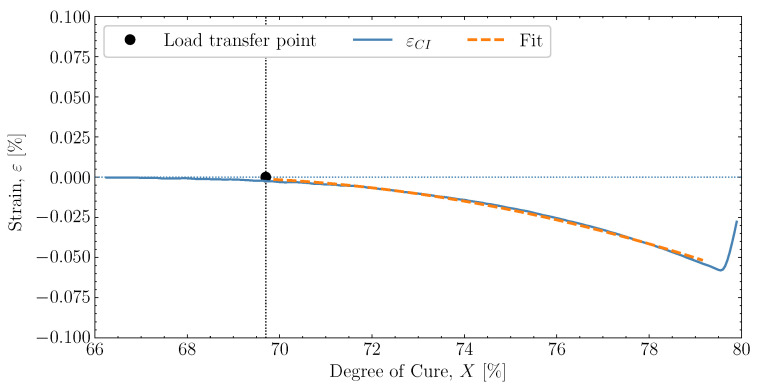
Fitting and extrapolation to determine the volumetric shrinkage based on strain measurements at pre-cure of $\left[50_{M}\right]80_{M}$. The blue line represents the strain measured, similar to Figure 3. The dashed orange line represents the fitted behaviour. The black dot represents the load transfer point, corresponding to $X_{\sigma }$.

To evaluate the pre-cure effects in a simulation context, it is necessary to quantify the volumetric shrinkage related to the chemical strain and the thermal expansion behaviour of the resin. The following will quantify these shrinkages to create the necessary inputs for the model. For quantifying the volumetric shrinkage, the experiment $\left[50_{M}\right]80_{M}$ (see Figure 8) is used to fit the load-transferring linear chemical strain during the initial pre-cure hold at the constant temperature of 50 °C. The $\left[50_{M}\right]80_{M}$ case was ideal, as the temperature changes are small and the cure temperature is far away from $T_{g}$ during the pre-cure, avoiding the vitrification effects from the glass transition.

The strain used to determine volumetric shrinkage is that measured from the load transfer point until just before the heat-up to the post-cure, which is combined with the predicted degree of cure using the cure kinetics model and the thermal history measured by the thermocouple. The temperature changes are so small in this region that the thermal contribution is assumed to have no influence. By applying the equation for the linear chemical strain (13) to the strain measured in this region as a function of degree of cure and substituting the incremental volumetric shrinkage with (4), the strain can be used to fit the evolution of the chemical shrinkage in the measured region. The parameters $X_{\sigma }$ and $X_{end}$ are input parameters, where $X_{\sigma }$ is the average from Figure 4 and $X_{end}$ is the maximum achievable, judged unlikely ever to exceed much more than 98%. Extrapolation from the fitted region can estimate the end value of the load-transferring volumetric shrinkage $V_{sh}^{end}$. By doing so, the extrapolated shrinkage is found to be −1.1%. This extrapolated value of $V_{sh}^{end}$ together with the values of $X_{\sigma }$ and $X_{end}$ are found in Table 3.

The total volumetric shrinkage from the liquid to the fully cured state is $V_{sh}^{tot}$ of −5.2% based on density measurements. The resin density in liquid state $\rho_{resin}^{init}$ was found using a liquid pycnometer, and the value was found to be $1088\;kg/m^{3}$, based on an average of three measurements. The cured density $\rho_{resin}^{end}$ was found using Archimedes principle on five samples cut from a cured panel with X>95%. The average value was found to be $1145\;kg/m^{3}$ and is judged to be fairly independent of the cure conditions [5]. Judging by the magnitude of the total shrinkage $V_{sh}^{tot}$, the load-transferring part $V_{sh}^{tot}$, induced from $X_{\sigma }$ until $X_{end}$, is considered reasonable, especially when compared with another study [19] estimating the load-transferring chemical shrinkage to be within −0.25% to −0.47%. The volumetric shrinkage fitted in this study would lead to a linear chemical strain of −0.36% when applying (13).

### 6.3. Fitting of Complex Thermal Expansion

A previously cured specimen, $\left[50_{M}\right]80_{M}$, was selected to fit the thermal expansion behaviour in a fully cured state. Before measuring the thermal expansion, the specimen was post-cured at 100 °C for 4 h to ensure no residual cure was left. According to a previous DSC analysis of this resin system, this should be sufficient to remove any residual cure of influence [17].

**Figure 9:**
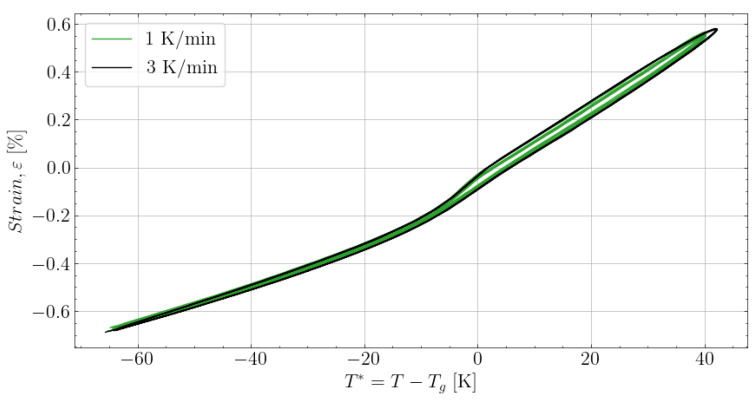
Strain measured based on a reheated $\left[50_{M}\right]80_{M}$ at both 1 K/min an 3 K/min after fully curing the specimen for 4 h at 100 °C.

The conditions selected to measure the thermal expansion were 1 K/min and 3 K/min. Figure 9 shows the measured strain response of $\left[50_{M}\right]80_{M}$ by heating up and cooling down three times with the selected rates. The negative magnitude strains observed at the start before heat-up are the cure-induced strain of $\left[50_{M}\right]80_{M}$. Heating with two different rates was observed to have no significant effects on the measurements. Furthermore, the continuous heating and cooling of the sample at both rates showed no noteworthy hysteresis.

**Figure 10:**
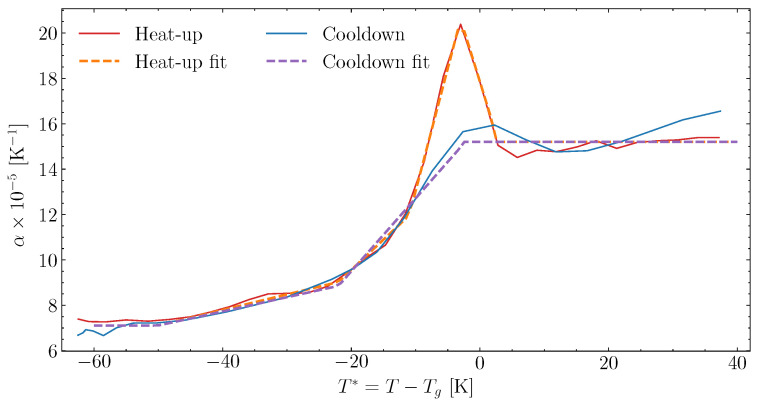
The thermal expansion evolution found by the derivative of the strain measured in Figure 9 for data measured at 1 K/min.

Based on the measured strain in Figure 9, the gradients can be found to fit the thermal expansion. The gradients are determined based on the 1 K/min data, segmented between heat-up and following cooldown. This is presented in Figure 10. The gradients of the measurements follow the behaviour described in Section 2.4 of the model proposed. The heat-up path is fitted to (6) and the cooldown to (7). The fitted thermal expansion in the glassy state $\alpha_{g}$ and the increase in thermal expansion $a_{1}$ to $a_{4}^{h}$ are listed in Table 4 together with the values of $T_{1}^{\prime }$ to $T_{5}^{\prime }$.

**Figure 11:**
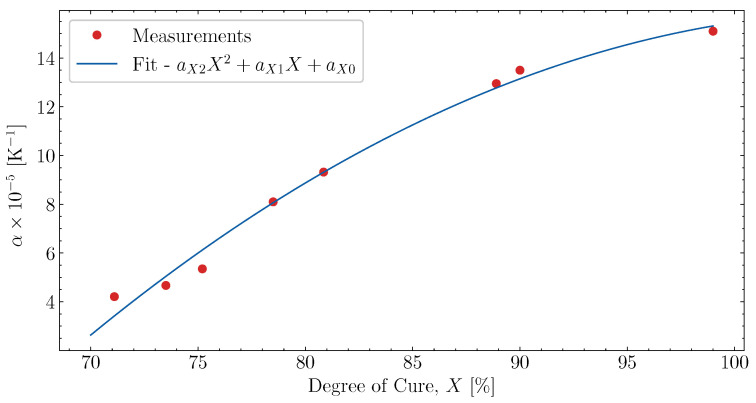
Evolution of thermal expansion for $T^{*}>T_{5}^{\prime }$ as function of *X*, for heating and cooldown.

For temperatures where $T^{*}>T_{5}^{\prime }$, the cooldown and heat-up expansion are constant relative to the influence of $T_{g}$. However, as the resin is curing, thermal expansion changes during curing above $T_{g}$ [8]. To determine the curing effect on thermal expansion, the cases listed in Table 2 have been measured during heat-up, past $X_{\sigma }$. The chemical shrinkage, based on the fit in Section 6.2, was subtracted from the measured cure-induced strain during the heat-up of the samples. The thermal expansion, $\alpha_{Xr}$, was then fitted to the linear gradient observed from the point where $T^{*}=T_{5}^{\prime }$ and until the heat-up ends. The fitted thermal expansion in the heat-up was correlated with the degree of cure, *X*, at the point where $T^{*}=T_{5}^{\prime }$, ignoring possible changes in the degree of cure over the fitted interval. In Figure 11, the measured thermal expansion values for the different cases are plotted together with the fit of (8). The second-order fit of the cure-dependent thermal expansion above $T_{g}$ seems to follow the measurements well. The fitted values are listed in Table 5. The function $\alpha_{Xr}$ will govern the cure-dependent thermal expansion during cooldown and heat-up, based on the little difference observed for the fully cured measurements in Figure 10.

## 7. Simulation of Cure Shrinkage

This section will predict the previously investigated cure-induced shrinkage with a simple 1D thermomechanical finite element model. The material models and behaviours described in Section 2 have been built into a modelling framework described in Section 5 and will be applied to elaborate on the material behaviour observed in Section 6.

### Model for the Thermal and Cure-Induced Strain Predicitions

**Figure 12:**
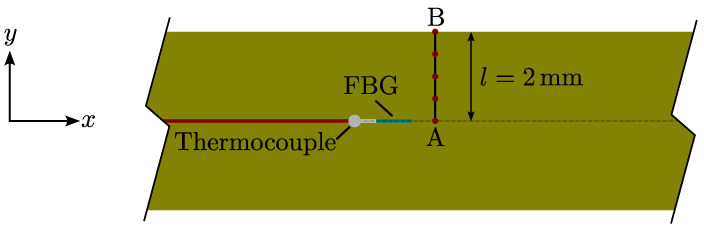
A cutout of the experimental setup showing the thermocouple and FBG. It illustrates how the 1D thermomechanical model is built to simulate the cure behaviour of the resin through the thickness.

The model for predicting the resin shrinkage is based on a finite element framework [24] and is a simple 1D thermomechanical model. Earlier work has shown that cure-induced strain can be captured with a material point model [21]. In this study, a similar approach is applied. However, to predict the thermal behaviour, the through-thickness response of the resin is required. This allows for the additional effect of the exothermal release of heat during the curing. The model is illustrated with Figure 12. Here, the resin bulk is illustrated in an xy-plane, with y as the principal model direction through the thickness. The region presented in Figure 12 is a narrow cutout of a vast resin bulk. The length and width of the domain are much larger than the thickness of the observed area, and possible thermal effects from possible edges can be ignored. Then, by only considering the thickness, the model stretches from the surface of the resin, called boundary B, to boundary A, in the middle of the resin. Imposing the conditions listed below:

A $\left(u_{y}\right)=\left(0\right)$, h=0 (symmetry of heat flow and displacement);
B $\left(u_{y}\right)=\left(free\right)$, $h=h_{c}$, $T=T_{oven}\left(t\right)$.

Boundary A is a symmetry condition for both the thermal and mechanical behaviour. As the resin is unconstrained, the model can contract in the y-direction. The heat flow *h*, from the surface of B, $h_{c}$, is the heat transfer coefficient enforced by the air movement possible from inside the oven. The temperature applied in this boundary, $T_{oven}\left(t\right)$, is the oven temperature measured for each case in Table 2. The thermal response of the simulation is monitored at boundary A, at the location corresponding to that of the thermocouple and the FBG sensor in the experiment. The strain produced in the simulation is evaluated at Boundary B.

**Figure 13:**
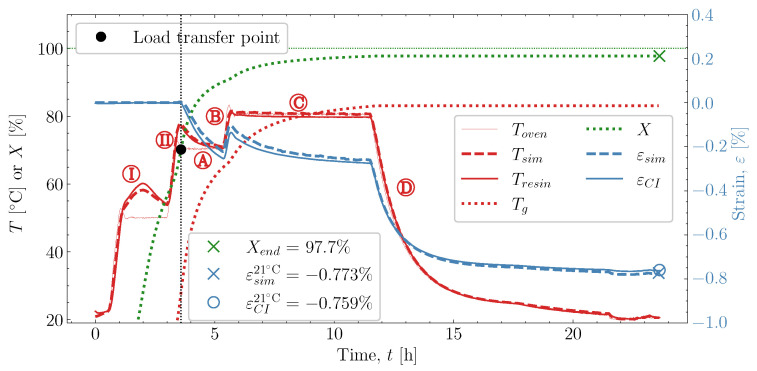
The predicted and measured values of temperature and strain over time for case $\left[50_{S}70_{S}\right]80_{M}$ as well as predicted *X* and $T_{g}$ by the simulation. The final values of the predicted and measured strain are shown in the plot.

For the prediction of the thermal behaviour, the necessary parameters are tabulated in Table 6. The total enthalpy of the reaction $H_{T}$ has been measured with DSC for the specific resin system [17]. The densities in Table 6, as reported in Section 6, have been determined experimentally. The heat capacity $c_{p,resin}$ and conductivity $k_{resin}$ were taken from [25,26], respectively. The convection coefficient for the air inside the oven has been taken from Carson et al. [27]. The cure-induced strain predicted by the model develops following the theory in Section 2. The primary components are the chemical and thermal strain, adding to the simulated cure-induced strain. In Figure 13, the simulation of the cure experiment $\left[50_{S}70_{S}\right]80_{M}$ is plotted, and the strain and temperature and strain from Figure 3 are included on top of the predicted strain and temperature by the model. In Appendix A, figures of the remaining cases for comparison based on the cases in Table 2 are compiled. To assist the description of the model behaviour, the following notation is used:

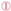
1. Isothermal— Pre-cure;
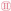
1. Ramp—Pre-cure;
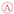
2. Isothermal—Pre-cure;
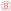
2. Ramp —Pre-cure;
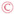
Isothermal—Post-cure;
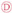
Cooldown after Post-cure.

**Table 6:** Thermal properties for the simulations.

$c_{p,\mathrm{resin}}$ [J/(kgK)]	$k_{r}$ [$W/(m^{2}K)$]	$H_{T}$ [$\frac{J}{\mathrm{kg}}$]	$\rho_{\mathrm{resin}}^{\mathrm{init}}$ [$\mathrm{kg}/m^{3}$]	$\rho_{\mathrm{resin}}^{\mathrm{end}}$ [$\mathrm{kg}/m^{3}$]	$h_{c}$ [$W/(m^{2}K)$]
1900 [25]	0.14 [26]	$4.7\;\times \;10^{5}$	1088	1145	15 [27]

The predicted temperature in the simulation results from the temperature load $T_{oven}\left(t\right)$, the thermal boundary condition and the cure kinetic behaviour. This results in the development of the noticeable exotherm during the two parts of the pre-cure, 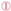 and 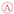. The predicted temperature by the simulation matches well with the monitored temperature from the thermocouple inside the resin. This is also observed in the remaining eight cases studied, found in Appendix A.

The strain predicted depends on the thermal behaviour, as the temperature, corresponding degree of cure *X*, and glass transition temperature $T_{g}$ are computed for every increment in the simulation. Once the load transfer point is reached, the incremental thermal and chemical strains develop. In Figure 13, the simulated cure-induced strain is predicted well. Both in terms of the shrinkage occurring during 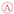, which is influenced heavily by the thermal and chemical strain occurring simultaneously. Followed by the heat-up 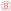, then the post-cure 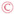 and the cooldown 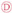, these also show good correlation between experiment and simulation. At the end of the cure, both the experimental observed cure-induced strain $\varepsilon_{CI}^{21^{\circ }C}$ and the simulated $\varepsilon_{sim}^{21^{\circ }C}$ are shown in Figure 13, as well as the final value of the simulated *X*. A comparison of the final simulated degree of cure with the final predicted one based on the thermocouple temperature monitored shown in Figure 3 is relevant. The differences are negligible; thus, the simulated cure development is accurate within the experimentally predicted. In terms of the differences observed between the simulated and measured strain, the deviation relative to the experiment was found to be within 2%. Hence, the simulation is overall satisfactory. The deviations and cure-induced strains observed for all the simulations and corresponding experiments are tabulated in Table 7. The overall deviation was found to be within 2–6% and the average deviation around 3%. With a simulation that matches the observed experimental behaviour well in all cases. The results from both experiments and simulations are available for download [23].

**Figure 14:**
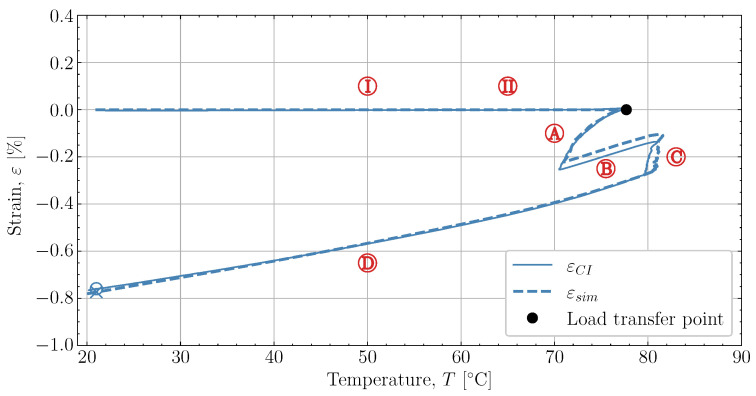
The predicted and measured values of strain as function of temperature for case $\left[50_{S}70_{S}\right]80_{M}$.

To better clarify how the thermal strain prediction affects the model behaviour during the curing, the experimental and simulated cure-induced strain is plotted in Figure 14 as a function of temperature. The figure demonstrates that the model predicts the cooldown during 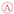 well, although it underestimates the shrinkage somewhat in magnitude. During the following heat-up 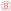, the expansion observed in the experiment is parallel with the expansion simulated. Therefore, the simulation can capture the expansion and contraction observed experimentally while curing progresses. This is important as the contraction and expansion occurring during 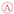 and 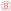 both occur well above $T_{g}$. This means that the expansion and contraction should be influenced by curing as per the thermal expansion model applied in Section 2.4.

**Figure 15:**
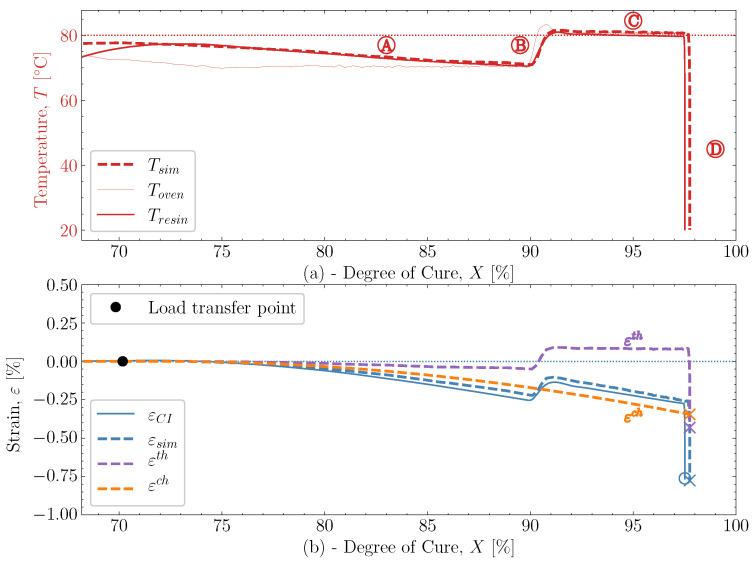
(**a**) The predicted and measured temperature in the resin as well as oven temperature as a function of the degree of cure. (**b**) The predicted and measured values of strain as a function of the degree of cure for case $\left[50_{S}70_{S}\right]80_{M}$.

The final cooldown 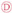 that occurs from 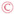 and down to room temperature is unaffected by any significant changes in *X* and demonstrates that the model can also capture the cured contraction well from just below $T_{g}$ and until far away from $T_{g}$. The simulated strain is plotted as a function of the degree of cure, *X*, against the experiment in Figure 15, where Figure 15a demonstrates the temperature development of the experiment $T_{resin}$, simulation $T_{sim}$ and the oven temperature $T_{oven}$ as a function of *X*. The temperatures of the experiment and simulation agree. There is a slight variation between the oven temperature and the resin temperatures.

This lag appears due to the heat flow through the thickness of the sample. Figure 15b demonstrates the experimental and simulation strains as a function of *X*. This makes it easy to distinguish the thermal strain from the chemical strain observed in the simulation. As the temperature drops during the pre-cure 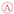, the simulated thermal strain is also observed to drop. The simulated chemical strain also decreases continuously as the degree of cure increases. It should be possible to check whether the chosen volumetric shrinkage model (4) adapted for the chemical strain matches the experimentally observed behaviour. The simulated strain $\varepsilon_{sim}$ is seen to under-predict the shrinkage occurring during slightly 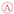, but follows in parallel with the experimental strain for the duration of 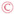. After that, the curing ends with the cooldown 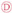. Even though there generally is this slight offset between experimental and simulated, the offset does not increase or decrease slightly. Indicating that the proposed shrinkage behaviour follows the experimental behaviour well. The simulations are, therefore, quite capable of determining the effects observed experimentally.

**Figure 16:**
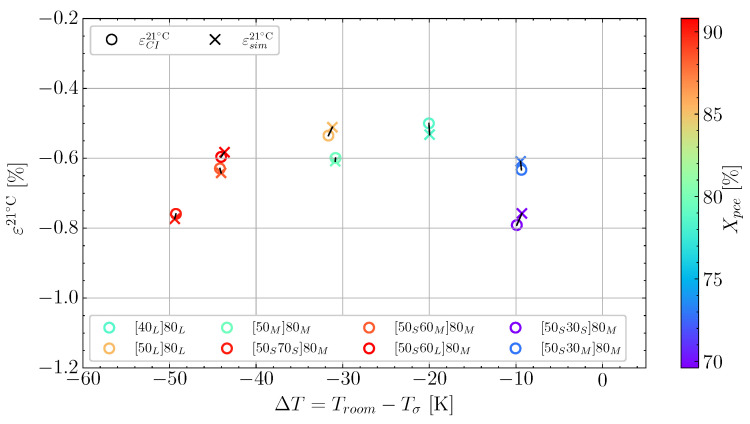
Comparison between the cure-induced strain from the experiments and simulations plotted together and linear and second-order tendencies plotted together. Circles indicate the measured shrinkages and the crosses represent the simulated shrinkage.

To demonstrate this graphically across the range of cases, Figure 16 shows an extended version of Figure 5. The final simulated and measured values of cure-induced strains are plotted together, and the possible differences are shown. The trendline adapted in Figure 5 is not applied here as the pre-cure has shown a high dependency on cure-induced strains. The fact that a very low achieved ΔT for case $\left[50_{S}30_{S}\right]80_{M}$ and $\left[50_{S}30_{M}\right]80_{M}$ results in high cure-induced strain signifies the dominating effect due to pre-cure and, more precisely, $X_{pce}$. This is attributed mainly to the thermal expansion model applied. Stressing that even though ΔT influences the level of cure-induced strain observed it is necessary to consider the complex thermal expansion to determine the cure-induced strain accurately.

**Figure 17:**
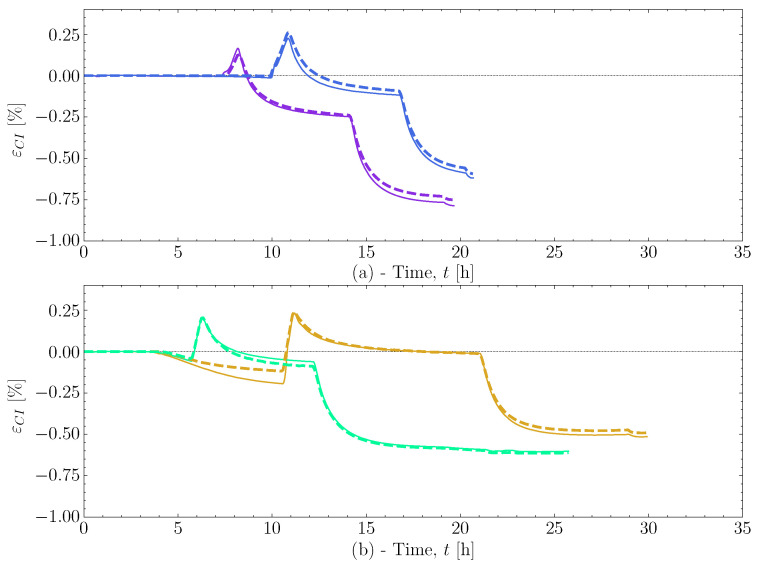
The comparison of pre-cure effects between the experiments with solid lines and simulations with dashed lines. The colours for the cases refer to the same colour bar for $X_{pce}$ in Figure 16. (**a**) The experiments and simulations of case $\left[50_{S}30_{S}\right]80_{M}$$\left[50_{S}30_{M}\right]80_{M}$. (**b**) The experiments and simulations of case $\left[50_{L}\right]80_{L}$ and $\left[50_{M}\right]80_{M}$.

Adapted in the simulation, this cure-dependent thermal expansion results in a similar low expansion during the heat-up for the cases $\left[50_{S}30_{S}\right]80_{M}$ and $\left[50_{S}30_{M}\right]80_{M}$, as observed in Figure 17a. Demonstrating that the simulation can capture the complex thermal expansion behaviour observed. The low thermal expansion at a lower degree of cure results in more shrinkage being transferred at the cooldown after post-curing. As simulated for the two cases, $\left[50_{S}30_{S}\right]80_{M}$ and $\left[50_{S}30_{M}\right]80_{M}$ agreed well with the measured strain. This limits the ability to reduce the cure-induced strain for ΔT from −20 K to 0 K for this specific resin system. At the other end of the ΔT axis in Figure 16, the cases $\left[50_{L}\right]80_{L}$ and $\left[50_{M}\right]80_{M}$ as well as $\left[50_{S}60_{M}\right]80_{M}$ and $\left[50_{S}60_{L}\right]80_{M}$ show that an increase in cure-induced strain is present. However, the difference in $X_{pce}$ between $\left[50_{M}\right]80_{M}$ and $\left[50_{L}\right]80_{L}$ is approximately 5% which, even though it is a more considerable difference than the 3.4% $\left[50_{S}30_{S}\right]80_{M}$ and $\left[50_{S}30_{M}\right]80_{M}$, the effect of ΔT is more dominating for higher values of $X_{pce}$. This is because the resin has cured significantly more; thus, the cure-dependent thermal expansion has developed much more, making the temperature at load transfer much more critical. This effect is reflected in the simulation and is due primarily to the implemented thermal expansion model. Therefore, the developed simulation can accurately predict the complex shrinkage observed over various experimental cases.

## 8. Conclusions

A specific epoxy resin system has been studied to quantify the cure-induced strain expected to develop during the curing in an unconstrained experimental setup. The cure-induced strains arising from various experiments were rather complex for the cure profiles investigated. The thermal expansion during the heat-up at the end of the pre-cure was cure-dependent and dependent on the glass transition temperature. A novel complex thermal expansion model and a model for the load-transferring volumetric shrinkage related to chemical cross-linking of the resin were proposed. The governing factors, such as chemical and thermal shrinkage leading to the experimentally observed cure-induced strain, could be quantified by fitting experimental observations to the proposed models. This was performed to investigate the ability of the proposed models to capture the behaviour of the cure-induced strain seen experimentally.

A simulation method was proposed to simulate the cure-induced strain across various cases accurately. The simulations correlated well with the experiments and agreed with the experimental observations, thus validating the simulation method. The simulations showed that the complex thermal expansion and the conventional volumetric shrinkage models were necessary for accurately predicting the cure-induced strain. The behaviour of the resin studied depended on the load transfer temperature and the development of the degree of cure at the pre-cure stage. This makes the complex thermal expansion model especially essential for accurately predicting cure-induced strains in simulations.

To lower the cure-induced strain in an unconstrained system, like the one investigated, it is henceforth essential to consider the investigated effects to minimise potential residual stresses in a setup, constraining the resin behaviour, and thus, inducing residual stresses. In a compact sense, a list can be drawn of the parameters necessary to make precise predictions of cure shrinkage:

- Perform DSC analysis to characterise the cure behaviour and determine the parameters for the cure kinetic model, glass transition temperature evolution, and the enthalpy of the reaction;
- Determine the load transfer initiation in the resin and determine/estimate the load transferring part of the volumetric shrinkage;
- Define the complex nature of the thermal expansion of the specific resin.

The listed parameters are the key aspects necessary to define the field to potentially minimise cure-induced strains from the curing of thermosetting epoxy resins. The parameters and material behaviour presented in this work can be further utilised in experiments where epoxy is mechanically constrained by surroundings. Leading to accurate predictions of the thermal and chemical behaviour for the build-up of residual stresses.

## Figures and Tables

**Table 1:** Parameters used for the prediction of *X* and $T_{g}$ of the specific resin system [17].

*A* [$s^{-1}$]	$e_{a}$ [kJ/mol]	*n* [-]	*m* [-]	*C* [-]	$X_{\mathrm{cT}}$ [K^−1^]	$X_{c0}$ [-]	$T_{g0}$ [°C]	ξ [-]	$T_{g\infty }$ [°C]
$2.50\;\cdot \;10^{5}$	56.24	1.83	0.41	43.7	$5.27\;\cdot \;10^{-3}$	−0.885	−42.0	0.487	89.0

**Table 2:** Cure profiles for investigations of cure-induced strains for an unconstrained resin. The ramps for heating are 1 K/min for all cases. Cooling ramps are approximately −0.1 K/min for cases with cooling during pre-cure. Each investigated case’s degree of cure results, and the final cure-induced strains measured at $T_{room}=21$ °C are given.

Cure ID	1. Pre-Cure	2. Pre-Cure	Post-Cure	$X_{\sigma }$	$X_{\mathrm{pce}}$	$X_{\mathrm{end}}$	$\varepsilon_{\mathrm{CI}}^{21^{\circ }C}$
	**[h @ °C]**	**[h @ °C]**	**[h @ °C]**	**[%]**	**[%]**	**[%]**	**[%]**
$\left[40_{L}\right]80_{L}$	12 @ 40	-	10 @ 80	71.6	78.5	98.3	−0.500
$\left[50_{L}\right]80_{L}$	10 @ 50	-	10 @ 80	68.9	85.2	98.2	−0.536
$\left[50_{M}\right]80_{M}$	5 @ 50	-	6 @ 80	67.7	79.2	97.3	−0.603
$\left[50_{S}70_{S}\right]80_{M}$	2 @ 50	2 @ 70	6 @ 80	66.8	90.0	97.5	−0.759
$\left[50_{S}60_{M}\right]80_{M}$	2 @ 50	4 @ 60	6 @ 80	70.8	88.3	97.7	−0.629
$\left[50_{S}60_{L}\right]80_{M}$	2 @ 50	8 @ 60	6 @ 80	72.8	90.3	97.8	−0.596
$\left[50_{S}30_{S}\right]80_{M}$	1.5 @ 50	3 @ 30	6 @ 80	69.6	69.6	97.4	−0.791
$\left[50_{S}30_{M}\right]80_{M}$	1.5 @ 50	6 @ 30	6 @ 80	69.7	72.9	97.4	−0.633

**Table 3:** Volumetric shrinkage for the Johnston shrinkage model determined for $\left[50_{M}\right]80_{M}$ [9]. $X_{\sigma }$ is the average taken from Figure 4 and $X_{end}$ is upper realistic achievable bound.

Cure ID	$X_{\sigma }$ [%]	$X_{\mathrm{end}}$ [%]	$V_{\mathrm{sh}}^{\mathrm{end}}$ [%]
$\left[50_{M}\right]80_{M}$	69.7	98.0	−1.1

**Table 4:** Parameters fitted for the thermal expansion model (6) and (7) based on FBG measurements of reheated cured specimen.

$\alpha_{g}$ [K^−1^]	$a_{1}$ [K^−2^]	$a_{2}$ [K^−2^]	$a_{3}^{h}$ [K^−2^]	$a_{4}^{h}$ [K^−2^]
$7.11\;\times \;10^{-5}$	$6.85\;\times \;10^{-7}$	$2.67\;\times \;10^{-6}$	$1.08\;\times \;10^{-5}$	$-9.19\;\times \;10^{-6}$
$T_{1}^{\prime }$ [**K**]	$T_{2}^{\prime }$ [**K**]	$T_{3}^{\prime }$ [**K**]	$T_{4}^{\prime }$ [**K**]	$T_{5}^{\prime }$ [**K**]
−50	−22	−11	−3	2.5

**Table 5:** The fitted parameters for the function $\alpha_{Xr}$ (8). This relation is only valid for values of $X>X_{\sigma }$.

$a_{X0}$ [K^−1^]	$a_{X1}$ [K^−1^]	$a_{X2}$ [K^−1^]
$-96.0\;\times \;10^{-5}$	$209.6\;\times \;10^{-5}$	$-98.1\;\times \;10^{-5}$

**Table 7:** End value of ε for experiments and simulations after the cure profiles evaluated at $T_{room}=21$ °C and the deviation.

Cure ID	$\left[40_{L}\right]80_{L}$	$\left[50_{L}\right]80_{L}$	$\left[50_{M}\right]80_{M}$	$\left[50_{S}70_{S}\right]80_{M}$	$\left[50_{S}60_{M}\right]80_{M}$	$\left[50_{S}60_{L}\right]80_{M}$	$\left[50_{S}30_{S}\right]80_{M}$	$\left[50_{S}30_{M}\right]80_{M}$
$\varepsilon_{CI}^{21^{\circ }C}$ [%]	−0.500	−0.536	−0.603	−0.759	−0.629	−0.596	−0.791	−0.633
$\varepsilon_{sim}^{21^{\circ }C}$ [%]	−0.532	−0.511	−0.608	−0.773	−0.642	−0.583	−0.758	−0.609
Dev. [%]	6	5	2	2	2	2	4	4

**Figure A1:**
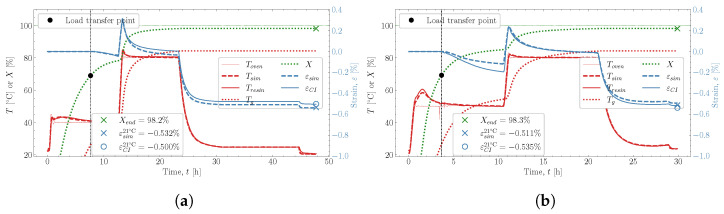
(**a**) Case $\left[40_{L}\right]80_{L}$ strain over time. (**b**) Case $\left[50_{L}\right]80_{L}$ strain over time.

**Figure A2:**
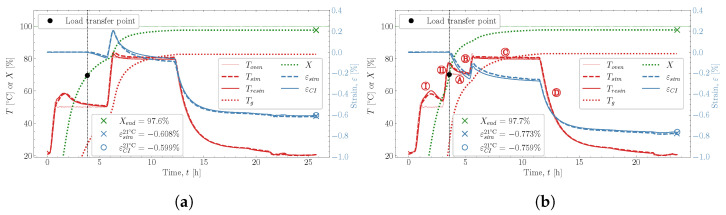
(**a**) Case $\left[50_{M}\right]80_{M}$ strain over time. (**b**) Case $\left[50_{S}70_{S}\right]80_{M}$ strain over time.

**Figure A3:**
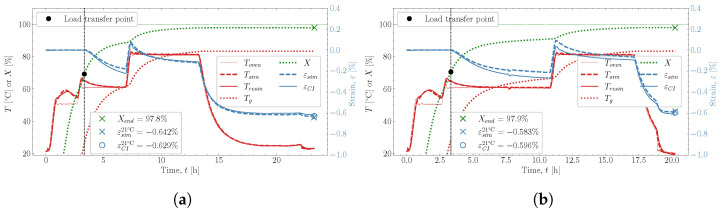
(**a**) Case $\left[50_{S}60_{M}\right]80_{M}$ strain over time. (**b**) Case $\left[50_{S}60_{L}\right]80_{M}$ strain over time.

**Figure A4:**
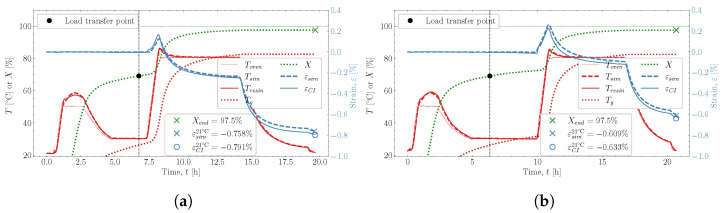
(**a**) Case $\left[50_{S}30_{S}\right]80_{M}$ strain over time. (**b**) Case $\left[50_{S}30_{M}\right]80_{M}$ strain over time.

## Data Availability

The data from the analysed experimental cure cases are made available, together with the model and scripts, building the analysis files for predicting the experimental behaviour investigated. The data are provided in ‘.csv’ format and the script for ABAQUS simulations consists of a model file ‘.cae’, a python script is provided making the necessary changes to the model ‘.py’, and a script is provided for submitting several simulations in a shell script for Linux-based clusters ‘.sh’ [23]. The subroutine for predicting curing is available [24].
